# Machine learning–based prognostic prediction and subphenotype identification in trauma-induced coagulopathy using early blood glucose trajectories

**DOI:** 10.3389/fendo.2026.1839373

**Published:** 2026-09-15

**Authors:** Guang-Yue Xu, Ying-Qi Zhang, Yan-Hong Yang, Qiao-Ling Lou, Xin-Liang Zhang

**Affiliations:** Department of Emergency Medicine, Hebei Medical University Third Hospital, Shijiazhuang, Hebei, China

**Keywords:** dynamic glucose trajectory, early risk stratification, prognosis, subphenotype, trauma-induced coagulopathy

## Abstract

**Background:**

Trauma-induced coagulopathy (TIC) is characterized by marked heterogeneity, and conventional classification methods have limited ability to capture its dynamic progression, frequently failing to account for inter-individual variability. This study aimed to develop a framework for identifying glucose trajectory phenotypes based on early blood glucose trajectories and to evaluate their association with 28-day mortality.

**Methods:**

A retrospective analysis was performed using data from 905 trauma patients in the discovery cohort obtained from the MIMIC-IV database and 260 trauma patients in a validation cohort from Hebei Medical University Third Hospital. Least absolute shrinkage and selection operator regression analysis was applied to identify prognostic variables, followed by the development and comparative evaluation of 12 machine learning models. Group-based trajectory modeling (GBTM) was used to characterize dynamic blood glucose trajectory patterns during the first 7 days following admission, and their associations with clinical outcomes were examined.

**Results:**

Six key prognostic variables were identified: red cell distribution width (RDW), white blood cell count, blood glucose, anion gap, lactate, and pH. The logistic classifier model achieved an area under the curve of 0.785 in the discovery cohort and 0.761 in the validation cohort, with acceptable calibration (ECE = 0.119). Among 184 eligible patients in the discovery cohort. GBTM identified three distinct blood glucose trajectory patterns: declining-then-stabilizing, persistently fluctuating, and stabilizing-then-rising. The stabilizing-then-rising trajectory pattern was significantly associated with the highest 28-day mortality (*p* = 0.034, adjusted OR 3.879, 95% CI 3.101-4.886, p < 0.001 in the pooled analysis, n = 1,165), a finding that was further confirmed in the validation cohort (*p* < 0.001).

**Conclusion:**

Application of GBTM to early blood glucose trajectories enables the identification of glucose trajectory phenotypes associated with differential mortality risk. This approach may provide a novel framework for dynamic risk stratification and targeted clinical management.

## 1 Introduction

Trauma constitutes a major global public health concern, accounting for approximately 1.35 million traffic-related mortalities annually, with nearly 93% occurring in low- and middle-income countries. Road traffic injuries are projected to become the seventh leading cause of death worldwide by 2030 (1). Among trauma-related fatalities, traumatic hemorrhage represents the most significant preventable cause of mortality, accounting for approximately 40% of cases and disproportionately affecting young individuals aged 5 to 29 years (1, 2). The pathophysiology of post-traumatic hemorrhage is complex and multifactorial. Severe trauma frequently precipitates trauma-induced coagulopathy (TIC), which is characterized by platelet consumption, depletion of coagulation factors, and activation of the fibrinolytic system (3). TIC is defined as a coagulation disorder occurring after trauma and is commonly identified by an international normalized ratio (INR) ≥ 1.5 or an activated partial thromboplastin time (APTT) ≥ 35 seconds (4, 5).

The underlying mechanisms of TIC are highly heterogeneous and involve multiple interacting pathways, including coagulation factor depletion, hyperfibrinolysis, endothelial injury, platelet dysfunction, and inflammatory responses (4–6). Following traumatic injury, tissue damage triggers the release of tissue factor, which activates the extrinsic coagulation pathway, leading to rapid consumption of coagulation factors, particularly factors V and VIII, as well as fibrinogen. Endothelial injury promotes the release of thrombomodulin, which activates the protein C pathway and subsequently inhibits coagulation processes (6, 7). In addition, trauma-associated hypoperfusion and acidosis further enhance fibrinolytic activity. This process is often reflected by elevated D-dimer levels and contributes to microvascular thrombosis and an increased risk of secondary bleeding (8, 9).

Importantly, TIC does not represent a single pathological entity but rather a bidirectional phenotypic spectrum that includes both hypercoagulable and hypocoagulable states. Hypercoagulability has been reported in approximately 22.2% to 85.1% of patients, whereas hypocoagulability occurs in approximately 25% to 35% of individuals with severe trauma. These phenotypes are associated with increased risks of thrombotic events and uncontrolled massive hemorrhage. Mortality increases approximately two- to four-fold and three- to four-fold in hypocoagulable states (4, 5, 10).

At present, the diagnosis and classification of TIC largely rely on static laboratory indicators measured at single time points. However, the physiological course of TIC is dynamic, typically evolving from an early hypocoagulable phase to a subsequent hypercoagulable state over hours to days. This phenotypic transition varies substantially among individuals and requires continuous monitoring for accurate characterization (11, 12). Consequently, traditional static laboratory parameters have a limited ability to differentiate TIC subtypes or to fully capture the complex pathophysiological processes involved, including fibrinogen depletion, platelet dysfunction, and hyperfibrinolysis (11, 13–15).

Current research on TIC primarily employs two methodological approaches: multi-parameter regression models based on conventional coagulation indicators and prediction models developed using machine learning algorithms. However, both approaches have a limited ability to characterize the dynamic evolution of TIC, particularly in distinguishing early hypocoagulable states from later hypercoagulable phases (16). Recent advances in continuous glucose monitoring (CGM) technology have revealed considerable intra-individual variability in fasting blood glucose levels among individuals without diabetes, thereby challenging traditional diagnostic paradigms based on static measurements.

In a large prospective cohort study, Shilo et al. reported that among 5,328 individuals with initially normal blood glucose levels, 40% and 3% were subsequently reclassified as prediabetic and diabetic, respectively, based on continuous monitoring data (17). These findings highlight the dynamic variability of blood glucose and underscore the potential value of longitudinal monitoring for improving metabolic assessment. This observation parallels the dynamic heterogeneity observed in TIC, in which patients may transition from early hypocoagulable bleeding to later hypercoagulable thrombosis. Conventional static indicators, such as prothrombin time (PT) and APTT, may therefore lead to misclassification of TIC phenotypes. To address this limitation, group-based trajectory modeling (GBTM) can be applied to longitudinal data to identify distinct temporal patterns.

In the present study, GBTM was applied to blood glucose measurements obtained during the first 7 days of intensive care unit (ICU) admission to identify potential glucose trajectory phenotypes and support precision-based interventions (18, 19). Traumatic stress may further influence glucose metabolism, as blood glucose trajectories may reflect trauma-induced insulin resistance and enhanced gluconeogenesis mediated by activation of the hypothalamic–pituitary–adrenal (HPA) axis (20, 21). These mechanisms provide a theoretical basis for early metabolic monitoring and intervention. Current clinical recommendations generally support maintaining blood glucose levels within a target range of 100 to 180 mg/dL, typically achieved through continuous intravenous insulin infusion.

The objectives of this study were twofold. First, to develop and validate dynamic glucose trajectory phenotypes using GBTM based on longitudinal blood glucose measurements during the 7 days after ICU admission. Second, to evaluate the clinical characteristics and outcomes associated with the identified subphenotypes, including 28-day mortality, in order to inform precision-oriented therapeutic strategies.

## 2 Methods

### 2.1 Study design and data sources

This study was conducted as a retrospective observational cohort study.

Data processing and statistical analyses were performed using DecisionLinnc v1.0.3.5, an integrated platform that supports data management, statistical analysis, and machine learning through a visual programming interface. Statistical significance was defined as a two-sided *p* value < 0.05.

The discovery cohort was derived from the MIMIC-IV database (version 3.1). Access to the database was granted to the author Guangyue Xu (certification number: 70272025). The study adhered to the principles of the Declaration of Helsinki and complied with established reporting guidelines for epidemiological observational studies.

The validation cohort consisted of trauma patients admitted to Hebei Medical University Third Hospital between January 2023 and December 2024. Data collection for the validation cohort was approved by the institutional ethics committee.

### 2.2 Study population

Patients were eligible for inclusion if they met the following criteria: (1) age ≥ 18 and < 80 years; (2) diagnosis of trauma with an Injury Severity Score (ISS) > 15 or equivalent criteria indicating severe trauma; and (3) ICU length of stay greater than 24 hours. Exclusion criteria included: (1) mortality within 24 hours of admission; (2) pre-existing coagulation disorders such as cirrhosis or malignancy; and (3) absence of key clinical or laboratory data required for analysis.

### 2.3 Variable extraction

Baseline data: Baseline characteristics included demographic information (age and sex) and vital signs recorded at admission.

Coagulation function indicators: Coagulation-related parameters included PT, INR, APTT, and platelet count. The first laboratory test results obtained after ICU admission were used.

Related indicators: Additional laboratory variables included WBC count, lactate, pH, and red cell distribution width (RDW). Blood glucose measurements consisted of the first morning glucose value recorded each morning during the first 7 days following ICU admission.

Primary outcome: The primary outcome was 28-day mortality.

This study aimed to develop a clinical predictive model based on routinely available clinical indicators. The discovery cohort derived from the MIMIC-IV database provided a comprehensive set of baseline variables, enabling detailed characterization of patient profiles. The validation cohort from Hebei Medical University Third Hospital collected core clinical and laboratory data through an electronic medical record system. Both cohorts contained all variables required for inclusion in the final predictive model. Although differences in laboratory reference ranges and measurement intervals existed between the U.S.-based discovery cohort and the Chinese validation cohort, analyses were performed using unstandardized raw values. Data collection procedures and measurement frequencies remained consistent between cohorts.

In the discovery cohort, feature selection techniques, including LASSO regression analysis, were applied to identify prognostically relevant variables from the full dataset (see the Feature Selection section for details). All selected variables were available in the validation cohort, ensuring the feasibility of external validation. The optimal predictive model developed in the discovery cohort (logistic classifier model) was subsequently applied to the validation cohort to calculate predicted risk scores. Model discrimination was evaluated using the area under the receiver operating characteristic curve (AUC), and model calibration was assessed using calibration curves.

### 2.4 Statistical analysis

#### 2.4.1 Data preprocessing

Variables with missing values below 20% were imputed using a random forest–based imputation method.

Considering that clinical sampling intervals were not equally spaced and the overall proportion of missing data was relatively low (less than 15% for each variable), we did not apply complex time-series imputation models that explicitly estimate temporal autocorrelation, such as state-space models or ARIMA models. Instead, we selected a simpler linear interpolation method to ensure clinical interpretability and reproducibility of the results. To validate this approach, we performed a complete-case sensitivity analysis by excluding all patients with missing glucose measurements to assess whether the imputation method influenced the trajectory classification results.

#### 2.4.2 Predictive model construction and feature selection:

##### 2.4.2.1 Feature variable selection

Normality testing was conducted for all continuous variables. Independent samples t-tests were applied to normally distributed variables, while non-normally distributed variables were analyzed using the Mann–Whitney rank-sum test. Categorical variables were compared using the chi-square test. These analyses were used to identify variables demonstrating significant group differences.

##### 2.4.2.2 LASSO regression (L1 regularization) analysis for feature selection and dimensionality reduction

The selected core features were incorporated into twelve machine learning algorithms—decision tree, gradient boosting tree, AdaBoost, LightGBM, logistic classifier, random forest, naive Bayes, CatBoost, XGBoost, support vector machine, perceptron classifier, and k-nearest neighbor classifier—to develop the predictive model. Variables identified through LASSO regression analysis were included as model inputs. Ten-fold cross-validation was applied to enhance model robustness and stability. The six variables selected by LASSO (i.e. RDW, WBC, glucose, anion gap, lactate, and pH) were used as the sole input features for all 12 machine learning algorithms. This parsimonious strategy was adopted to: (1) enhance clinical interpretability by focusing on routinely available emergency department laboratory parameters; (2) facilitate external validation in cohorts with limited variable availability; and (3) reduce the risk of overfitting, particularly for nonlinear models, given the relatively small sample size and class imbalance in the discovery cohort. To empirically assess whether this dimensionality reduction resulted in the loss of meaningful predictive information, sensitivity analyses were performed comparing the parsimonious six-variable models with full-feature models incorporating all available candidate variables, using both the logistic classifier and the GBDT algorithm (see Results section). All 12 machine learning models underwent hyperparameter optimization using grid search combined with 10-fold stratified cross-validation. The tuning process was conducted exclusively on the training set (70%, n=634), while the validation set (10%, n=90) was used for early stopping and model selection to prevent overfitting. The independent hold-out test set (20%, n=181) remained completely “locked” and was used only for the final unbiased evaluation of model performance.

For models with larger hyperparameter spaces, such as random forest and XGBoost, a staged grid search strategy was applied: an initial coarse search was performed across a broad range of parameter values to identify the approximate optimal region, followed by a fine search within the narrowed parameter range. All models underwent hyperparameter optimization using the same number of cross-validation folds (10-fold) and the same evaluation metric (PR-AUC), ensuring comparable training and evaluation conditions across algorithms. The specific hyperparameter search ranges and the final optimal configurations for each model are provided in **Supplementary Table 7**.

Model development (discovery cohort): All machine learning models were trained using the training set of the MIMIC-IV discovery cohort (70%, n=634). The validation set (10%, n=90) was used for hyperparameter optimization and early stopping to prevent overfitting. The independent hold-out test set (20%, n=181) remained “locked” and was used only for the final unbiased evaluation of internal model performance. LASSO feature selection was performed exclusively on the training set, with the optimal λ value determined through 10-fold cross-validation.

External validation: The final selected logistic classifier model, incorporating the six LASSO-selected variables with fixed regression coefficients and intercept, was treated as a “locked model” and directly applied to the independent external validation cohort (Hebei Medical University Third Hospital, n=260). No model retraining, parameter adjustment, or feature re-selection was performed using the external validation data. The external validation cohort remained completely independent from the model development process and was used solely to assess the model’s generalizability in a new patient population.

Model selection was based on the area under the receiver operating characteristic (ROC) curve (AUC). Model development was conducted using the training dataset, followed by evaluation in both internal and external validation cohorts. The entire study cohort was randomly stratified and divided into three independent subsets: a training set (70%, n=634), a validation set (10%, n=90), and a hold-out test set (20%, n=181). The training set was used for model training and 10-fold cross-validation; the validation set was used for hyperparameter optimization and early stopping to prevent overfitting; and the hold-out test set remained completely unused during model development and was reserved exclusively for the final unbiased evaluation of the selected optimal model. To prevent data leakage, all patient-level data were partitioned based solely on the unique patient identifier (subject_id), ensuring that all records from the same patient were assigned exclusively to a single dataset. Given that the MIMIC-IV database may contain multiple ICU admissions for the same patient, patient-level stratification was performed to ensure that records from the same individual were not distributed across different datasets. Model performance was assessed using ROC-AUC, sensitivity, and specificity. Decision curve analysis (DCA) and calibration curves were generated to evaluate potential clinical utility. Given the imbalance in outcome events within the discovery cohort (693 survivors vs. 212 non-survivors), the PR-AUC was primarily adopted as the principal metric for model selection and performance comparison. This was because PR-AUC provides a more reliable assessment of a model’s ability to discriminate the minority class (28-day mortality) in imbalanced datasets. The ROC-AUC, calibration curves (including expected calibration error, ECE), and DCA were used as complementary evaluation metrics to comprehensively assess model discrimination, calibration, and clinical utility. In addition, SHapley Additive exPlanations (SHAP) were used to quantify the contribution of individual features to prediction outcomes. A SHAP summary plot was produced to illustrate overall feature importance, while SHAP analyses of selected cases were performed to quantify the contribution of each feature to individual predictions, thereby facilitating interpretation of the model’s decision-making process. To enhance model interpretability, we applied the SHAP (SHapley Additive exPlanations) method to interpret the final selected logistic classifier. SHAP analysis was performed based on the final model constructed using the six LASSO-selected variables and quantified the marginal contribution of each feature to individual prediction outcomes. Feature importance was ranked according to the mean absolute SHAP value across all samples.

#### 2.4.3 GBTM construction

##### 2.4.3.1 Trajectory indicators

The trajectory variable was defined as the first morning glucose measurement obtained each morning between 06:00 and 08:00 during the first 7 days following ICU admission. For missing daily glucose measurements with a missing proportion below 15%, a random forest imputation method was applied.

The rationale for selecting the first fasting blood glucose measurement obtained between 06:00 and 08:00 as the trajectory variable was as follows: (1) this time window represents the routine morning blood sampling period in the ICU, with the highest data availability and standardization, thereby minimizing random variations caused by short-term disturbances such as nutritional infusion and temporary insulin administration; (2) morning steady-state glucose reflects the relatively balanced metabolic state achieved after overnight therapeutic adjustments and therefore provides greater clinical interpretability than measurements obtained at random time points; and (3) glucose measurements in the MIMIC-IV database were collected at highly irregular time intervals, and inclusion of all available time points could introduce substantial temporal heterogeneity and obscure clinically meaningful overall trends.

Before GBTM modeling, glucose measurements from different datasets (MIMIC-IV and the local validation cohort) were standardized using Z-score standardization. The standardization formula was: Z = (X − μ)/σ, where X represents the original glucose value (mmol/L), μ represents the mean glucose value within each dataset, and σ represents the corresponding standard deviation. The standardized glucose values were expressed as dimensionless scores with a mean of 0 and a standard deviation of 1.

The rationale for applying Z-score standardization was as follows: (1) the two datasets differed systematically in glucose measurement instruments, calibration methods, and reference ranges; therefore, directly using raw glucose values for cross-database trajectory modeling could introduce measurement-related bias as a confounding factor; (2) within a machine learning framework involving multiple features with different measurement scales, standardization is a conventional preprocessing step to ensure that feature contributions are not influenced by their original units; and (3) the R package gbmt used for GBTM modeling applies standardization by default to minimize the impact of differences in measurement scales when comparing trajectories across indicators.

The standardized trajectory values represent the degree of deviation of glucose levels from the dataset-specific mean, expressed in units of standard deviation. For example, a one-unit increase in the standardized value corresponds to an increase of one standard deviation in the original glucose concentration.

Patients were eligible for trajectory analysis if they had less than 15% missingness in daily morning glucose measurements (06:00–08:00) during the first 7 days after ICU admission. Among eligible patients (n = 184), missing individual daily glucose values were imputed using a random forest-based imputation method. The remaining 721 patients were excluded from trajectory analysis because complete 7-day morning glucose profiles could not be generated; however, these patients remained included in the development and validation of the machine learning prognostic models (**Supplementary Table 6**).

##### 2.4.3.2 Modeling and model selection

GBTM was applied to longitudinal blood glucose data from the training cohort. Models consisting of one to five trajectory subgroups were sequentially fitted. The optimal model was determined according to the following criteria: (1) Akaike Information Criterion (AIC), with the model demonstrating the lowest AIC among candidate models with two to five subgroups selected; (2) average posterior probability (AvePP) ≥ 70% for all subphenotypes; (3) each subgroup representing more than 5% of the total cohort; and (4) trajectory patterns demonstrating clinical interpretability.

During the GBTM modeling process, specific therapeutic interventions, such as insulin dosage and nutritional support strategies, were not included as covariates. This decision was based on the following considerations: the objective of trajectory classification in this study was to identify the overall patterns of metabolic evolution under real-world clinical management conditions, rather than to isolate treatment effects and derive a “pure” physiological signal. As a composite marker reflecting the interaction between the patient’s physiological state and clinical interventions, the glucose trajectory inherently incorporates the effects of treatment and therefore serves as an integrated predictor of clinical outcomes. Over-adjustment for intervention-related variables could artificially “purify” the trajectory phenotype and consequently reduce its predictive value in real-world clinical settings.

##### 2.4.3.3 Final model and subgroup matching

Based on these criteria, the model comprising three trajectory subgroups achieved the optimal balance between AIC and clinical interpretability. All subgroups demonstrated AvePP > 0.999, indicating extremely high classification accuracy. This model was therefore selected as the final model. Each patient was subsequently assigned to the trajectory subgroup corresponding to the highest posterior probability, generating a categorical variable termed “early blood glucose trajectory subphenotype.”

It should be emphasized that the glucose trajectories constructed in this study were not intended to represent a pure physiological stress response, but rather a clinical steady-state glycemic phenotype. This phenotype reflects the combined effects of the patient’s endogenous metabolic status (e.g. stress-induced hyperglycemia and insulin resistance) and clinical interventions (e.g. insulin infusion, nutritional support, and fluid resuscitation). In the real-world ICU setting, glucose levels are never a single isolated physiological signal but rather a product of the interaction between the patient’s condition and therapeutic management. Therefore, we interpreted the morning steady-state glucose trajectories as an integrated marker reflecting the dynamic evolution of metabolic status under routine clinical care, rather than an attempt to isolate a purely pathophysiological signal. Because the trajectory classes were derived exclusively from longitudinal blood glucose measurements, they are referred to as glucose trajectory phenotypes throughout this manuscript and should not be interpreted as directly measured coagulation phenotypes.

#### 2.4.4 Subphenotype characteristics and outcome association analysis

##### 2.4.4.1 Comparison of subgroup baseline characteristics

Baseline characteristics among the trajectory subgroups were compared using the Kruskal–Wallis test for continuous variables and the chi-square test for categorical variables.

##### 2.4.4.2 Outcome association analysis

A multivariable logistic regression model was used to evaluate the independent association between blood glucose trajectory subphenotypes and mortality risk, with “blood glucose trajectory subphenotype” included as the primary independent variable. Covariates were selected based on clinical relevance and the results of univariate analysis (*p* < 0.1). The final model included age, WBC, and lactate levels to adjust for the potential influence of baseline confounders on the outcome.

Validation analysis: In the local validation cohort, trajectory parameters derived from the MIMIC cohort were applied to reassign patient trajectory subtypes, and comparisons of the aforementioned baseline characteristics and outcomes were repeated.

A *p*-value < 0.05 was considered statistically significant.

#### 2.4.5 Statistical control for baseline differences between cohorts

Potential differences in population characteristics and treatment environments between the discovery cohort (MIMIC-IV, USA) and the external validation cohort (China) were acknowledged. To assess the possible influence of these differences on model evaluation, the following statistical control strategies were applied in all analyses involving model validation and cross-cohort comparisons:

##### 2.4.5.1 Main analysis (covariate adjustment)

During evaluation of predictive model performance in the validation cohort, the categorical variable “cohort origin” (discovery cohort vs. validation cohort) was included as a covariate within the model evaluation framework. Specifically, when calculating model discrimination (AUC) and generating calibration curves, the model was first fitted using the pooled dataset (discovery and validation cohorts combined), with “cohort origin” incorporated as a predictor variable. Model performance was subsequently assessed within the validation cohort subset. This approach enabled adjustment for cohort effects prior to comparison.

##### 2.4.5.2 Adjustment in multivariate models

During logistic regression analyses evaluating the association between blood glucose trajectory subtypes and mortality, both “cohort origin” and potential baseline confounders (WBC and lactate) were included as covariates. This adjustment ensured that the observed associations were independent of baseline differences between cohorts.

Sensitivity analysis (stratified analysis): Stratified analyses were conducted in which the original model performance was reported separately for the discovery and validation cohorts to allow direct comparison.

#### 2.4.6 Model calibration evaluation methods

Model calibration was evaluated using calibration curves and expected calibration error (ECE). The calibration curve illustrates the relationship between predicted mortality risk and observed mortality rates, thereby providing a visual assessment of model calibration performance across the full range of predicted risk. To quantify the precision of these estimates, 95% confidence intervals (CIs) for the ECE and for the DCA net benefit at representative threshold probabilities (0.1, 0.2, and 0.3) were calculated using 1,000 bootstrap resamples.

### 2.5 Study technical flowchart

The overall technical workflow of the study is presented in **Figure 1**.

**Figure 1:**
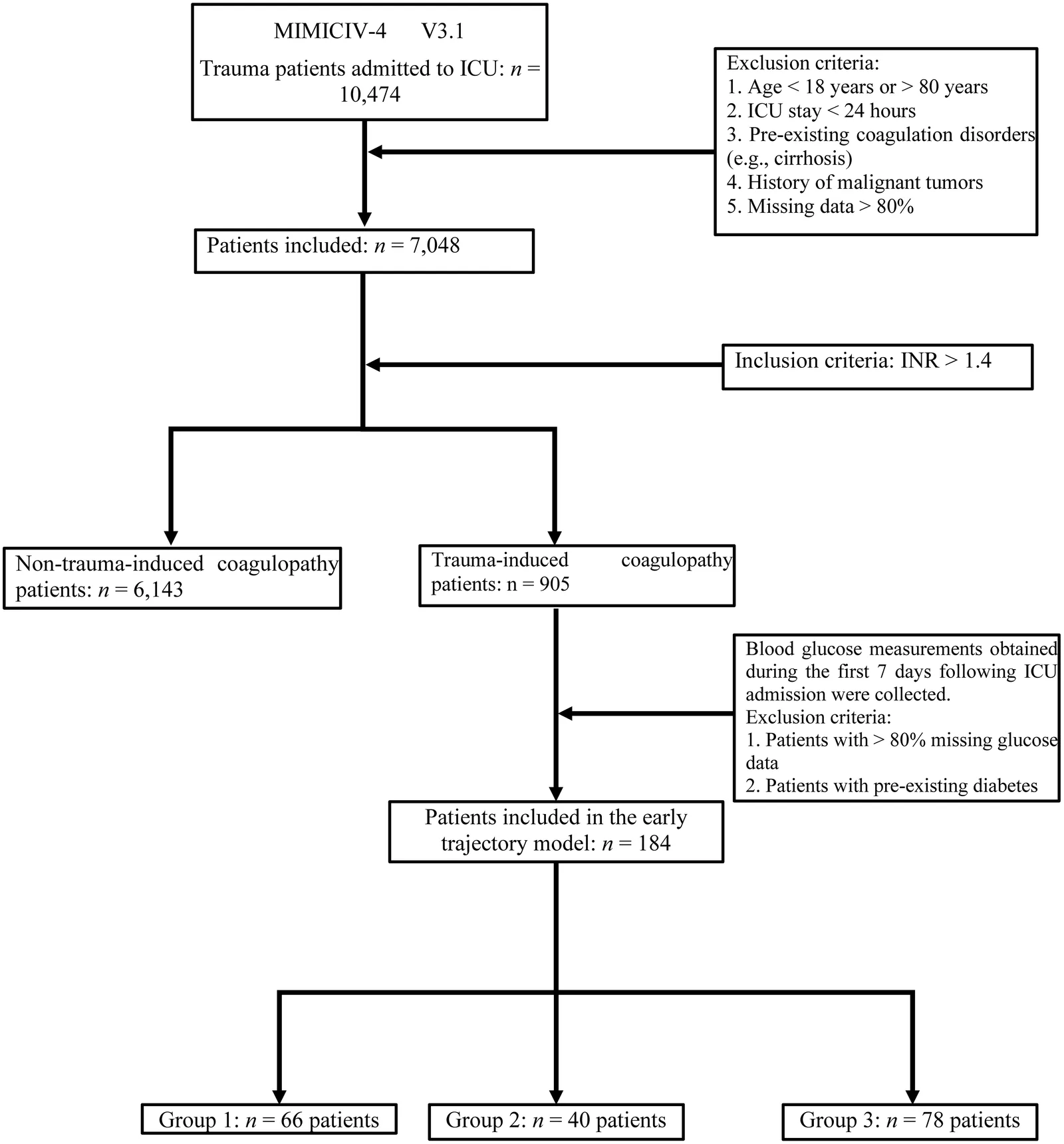
Study flowchart of patient selection and inclusion.

## 3 Results

### 3.1 Baseline characteristics of the MIMIC cohort and the local cohort

A total of 905 patients from the MIMIC database were included in the analysis, including 693 survivors (**Supplementary Table 1**). Compared with the non-survivor group, patients in the survivor group demonstrated lower heart rate, higher invasive and noninvasive systolic blood pressure, lower WBC count, lower absolute neutrophil counts, lower albumin levels, lower anion gap, lower blood glucose levels, lower total CO_2_, lower pH, and lower lactate levels. In the local cohort (**Supplementary Table 2**), among 260 patients, the survivor group was relatively younger and presented with higher pH, lower heart rate, lower WBC count, lower albumin levels, lower blood glucose levels, and lower lactate levels.

### 3.2 Feature selection

LASSO regression analysis was applied to identify relevant predictors in the training dataset (**Figure 2**). The variation in regression coefficients for the candidate variables is presented in **Figure 2A**, while the iterative analysis based on 10-fold cross-validation is presented in **Figure 2B**. Six variables were identified as being strongly associated with prognosis: RDW, WBC, blood glucose, anion gap, lactate, and pH. All six variables included in the final predictive model were available in the external validation cohort.

**Figure 2:**
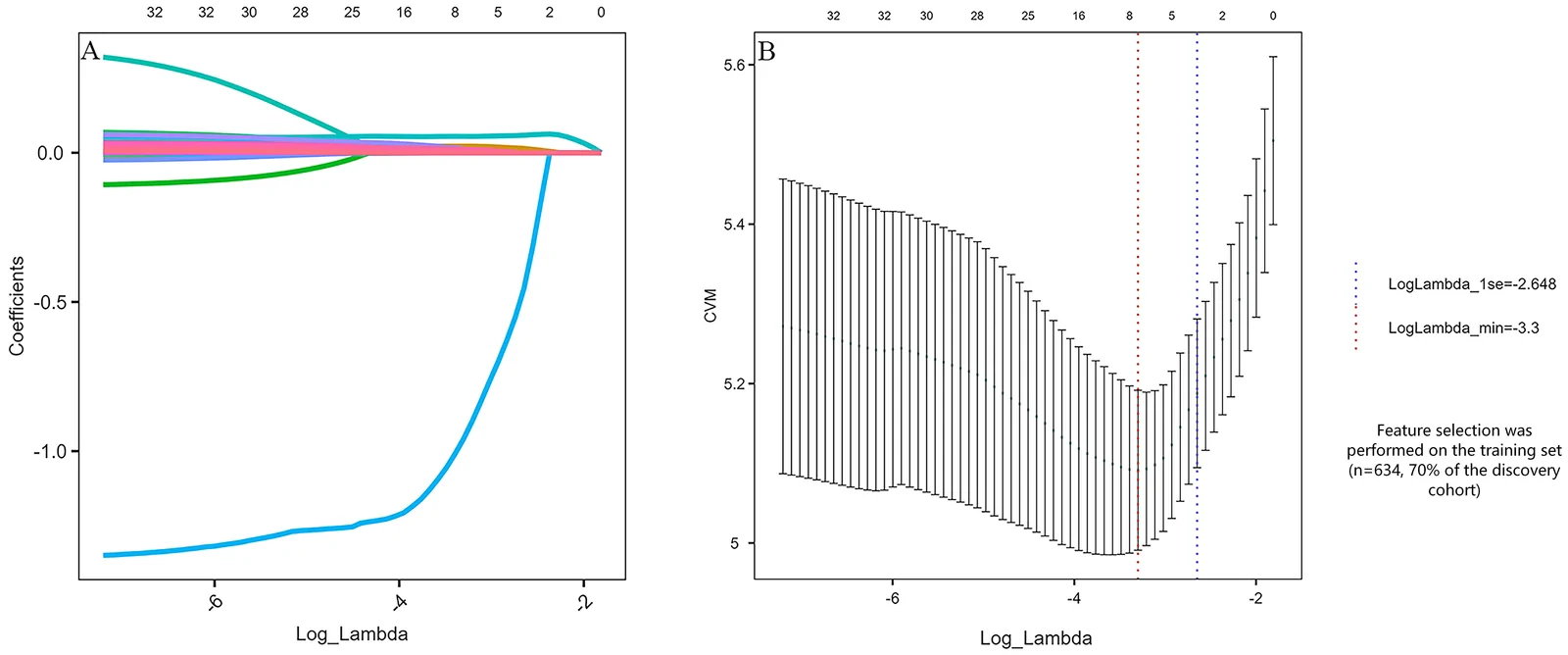
LASSO-based feature selection. Feature selection was performed on the training set (n=634, 70% of the discovery cohort) Panel **(A)** presents the coefficient trajectories of variables during LASSO feature selection; Panel **(B)** presents the optimal λ value determined by 10-fold cross-validation in the LASSO regression model.

### 3.3 Model performance comparison

The final performance of the optimal model was evaluated on the independent hold-out test set (n=181, 20% of the discovery cohort).

Twelve machine learning models were developed to predict mortality risk among patients with TIC in the ICU. **Figure 3A** illustrates the discriminative performance of these models based on ROC curves. All models demonstrated prognostic predictive ability, with the gradient boosting decision tree (GBDT) model achieving the highest performance. Based on ROC-AUC values, the 12 models were categorized into three performance tiers. The first tier (best-performing group) included the GBDT model, which achieved the highest AUC (AUC = 0.796), followed by the Logistic classifier, KNN, and LGBM models, all of which achieved an AUC of 0.786. The second tier (intermediate-performance group) consisted of CatBoost (AUC = 0.749), random forest (RF; AUC = 0.747), AdaBoost (AUC = 0.746), multilayer perceptron (MLP; AUC = 0.727), and decision tree (AUC = 0.720). The third tier (lower-performance group) included naïve Bayes (AUC = 0.686), support vector machine (SVM; AUC = 0.623), and XGBoost (AUC = 0.617). Considering the class imbalance in the dataset, we further evaluated model performance using precision-recall (PR) curves. The Logistic classifier achieved the highest PR-AUC (0.651), followed by GBDT (0.631), KNN (0.629), and LGBM (0.618). After integrating PR-AUC performance, calibration performance (ECE = 0.119), and clinical utility assessed by decision curve analysis (DCA), we ultimately selected the Logistic classifier as the optimal predictive model.

**Figure 3:**
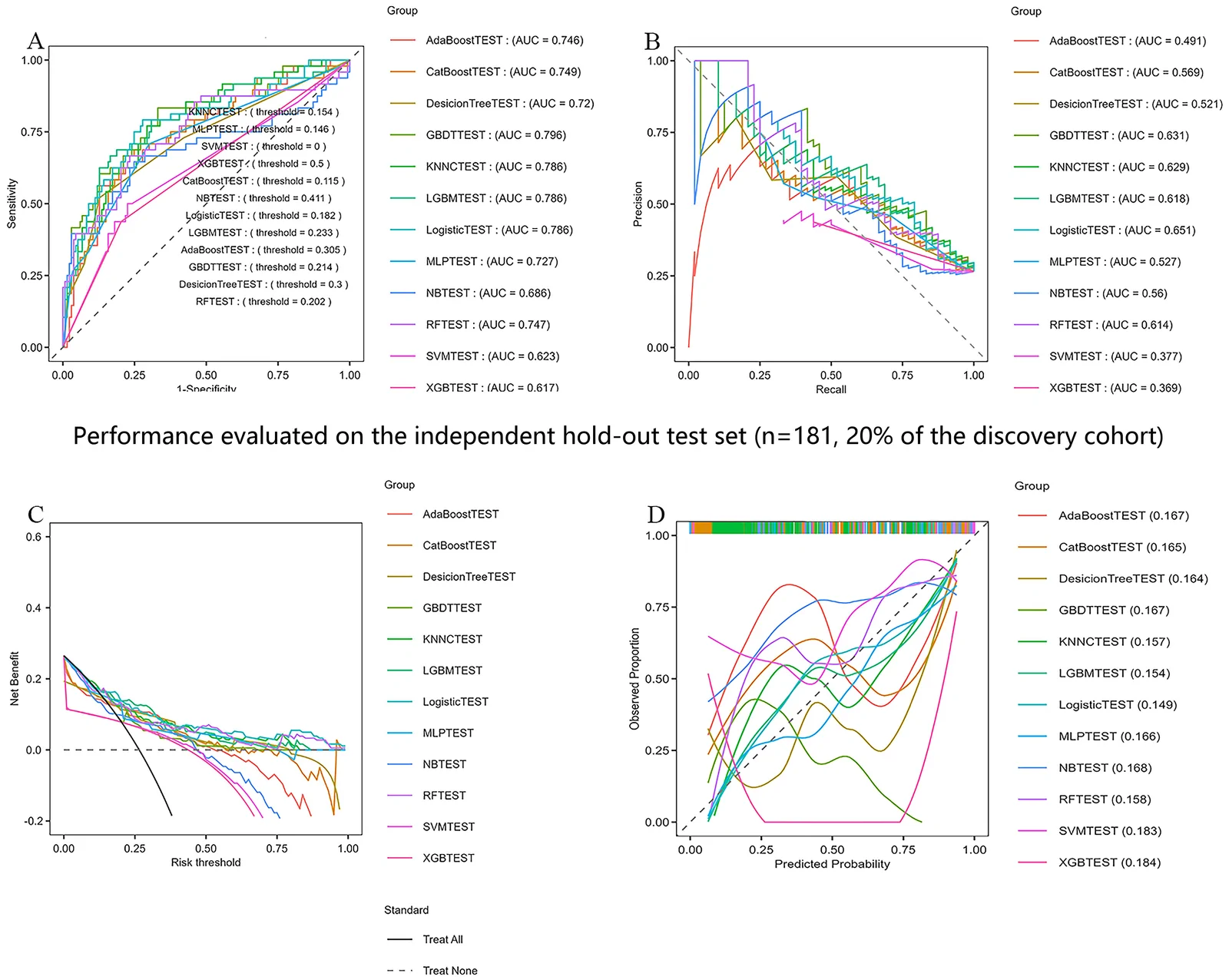
Performance, calibration, and clinical utility of the twelve machine learning models evaluated on the independent hold-out test set (n=181). Panel **(A)** presents the ROC curves; Panel **(B)** presents the PR curves; Panel **(C)** presents the DCA curves; Panel **(D)** presents the calibration curves.

Due to the imbalance between the number of survivors and non-survivors in the MIMIC database, precision–recall (PR) curves were additionally used to evaluate model performance (**Figure 3B**). Analysis of the relationship between precision and recall identified the logistic classifier model as the optimal model, achieving an AUC of 0.651. The GBDT (AUC = 0.631), KNN (AUC = 0.629), and LGBM (AUC = 0.618) models followed closely and outperformed the remaining algorithms.

Given the class imbalance in the dataset, PR-AUC was used as the primary criterion for model selection. As shown in **Figure 3B**, the Logistic classifier achieved the highest PR-AUC (0.651), outperforming GBDT (0.631), KNN (0.629), and LGBM (0.618). In addition, the Logistic model demonstrated good calibration performance (ECE = 0.119, **Figure 3D**) and achieved the greatest clinical net benefit in decision curve analysis (**Figure 3C**). Considering the priority of PR-AUC, calibration performance, and clinical applicability, the Logistic classifier was selected as the final recommended model.

To evaluate whether restricting the model input to the six LASSO-selected variables affected predictive performance, we compared the six-variable parsimonious model with a full-feature model incorporating all 24 candidate variables using both the Logistic classifier and the GBDT algorithm in the discovery cohort. The full-feature Logistic model achieved an AUC of 0.786, while the six-variable Logistic model also achieved an AUC of 0.786, with no statistically significant difference (DeLong test, *p* = 0.43). Similarly, the full-feature GBDT model (AUC = 0.784) did not demonstrate improved performance compared with the six-variable GBDT model (AUC = 0.796; *p* = 0.38). Calibration curves and decision curve analyses showed comparable performance between the parsimonious and full-feature models. These findings indicate that the six LASSO-selected variables captured the majority of prognostic information, and that the parsimonious modeling strategy did not compromise predictive performance (**Supplementary Figures 2**–**6**).

The clinical utility of the predictive models across different decision thresholds was further assessed using DCA (**Figure 3C**), allowing evaluation of their potential value in clinical decision-making. The horizontal axis of the DCA curve represents the decision threshold, whereas the vertical axis represents the net benefit. Net benefit reflects the overall clinical utility of model predictions by accounting for trade-offs at different threshold probabilities.

Calibration curves for the twelve models are presented in **Figure 3D**, illustrating their predictive reliability. Among the evaluated models, the logistic classifier model demonstrated strong agreement between predicted probabilities and observed outcomes.

Regarding clinical applicability, all models demonstrated substantial net benefit across a broad range of threshold probabilities (**Supplementary Table 3**), with the logistic classifier model achieving the greatest net benefit. Based on these findings, the logistic classifier model was selected as the optimal model for predicting TIC prognosis.

Q–Q plots (**Supplementary Figure 1**) were used to evaluate data distribution, indicating that the logistic classifier model exhibited normally distributed residuals, suggesting favorable model quality.

### 3.4 External validation

Despite baseline differences between the survival and mortality groups, the predictive model demonstrated strong generalizability. In the external validation cohort, the AUC of the ROC curve (**Figure 4A**) was 0.761 (95% CI: 0.676–0.846). Model calibration was assessed using a calibration curve (**Figure 4B**), which demonstrated good agreement between predicted and observed risks across most probability intervals. Calibration curves were selected instead of the Hosmer–Lemeshow test due to the latter’s sensitivity to sample size, whereas calibration curves provide a more intuitive assessment of model performance across different risk intervals. Quantitative assessment indicated that the ECE was 0.119, indicating some deviation between predicted probabilities and observed outcomes; however, the predictions retained practical clinical value.

**Figure 4:**
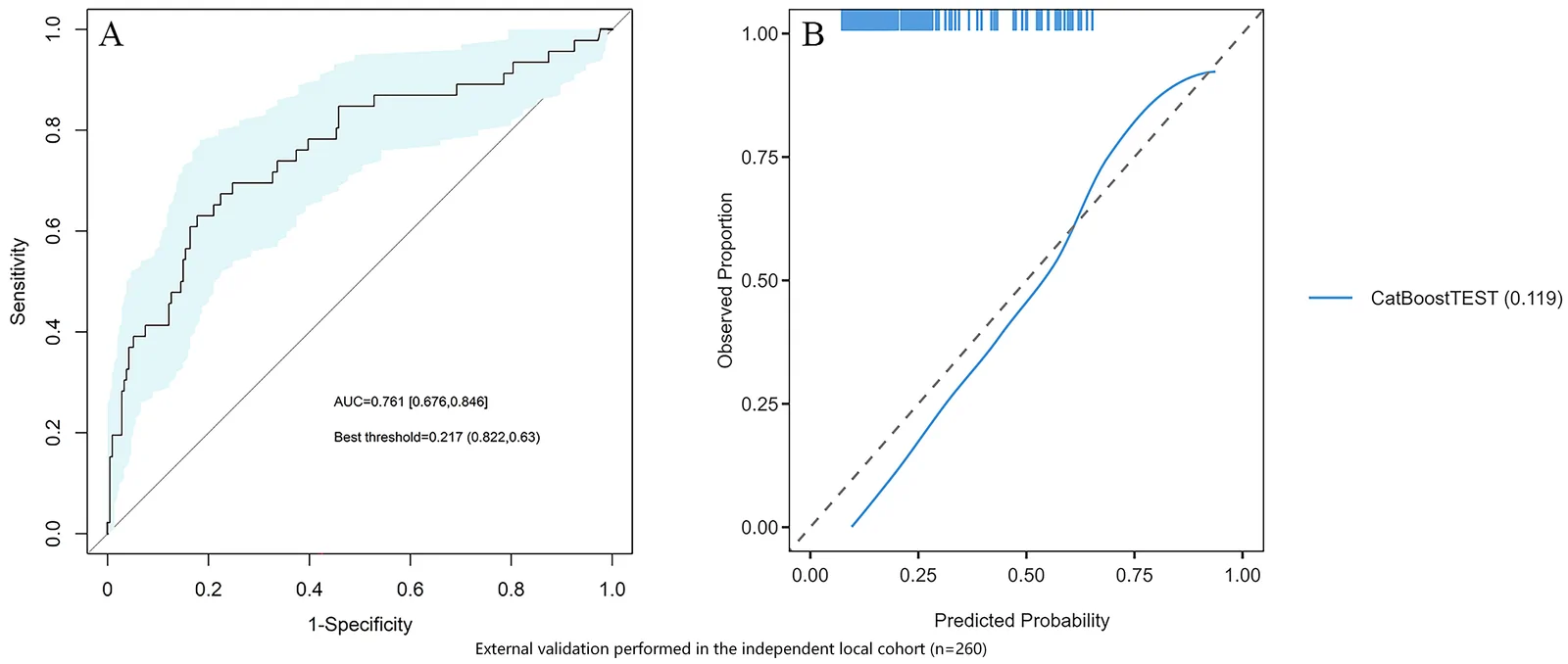
External validation of model performance and calibration. External validation performed in the independent local cohort (n=260) Panel **(A)** presents the ROC curve of the external validation cohort; Panel **(B)** presents the calibration curve.

### 3.5 Interpretability analysis

**Figure 5A** presents a beeswarm summary plot of the variables included in the logistic classifier model. The horizontal axis represents the SHAP value, while the vertical axis lists features ranked by cumulative SHAP importance. Each point corresponds to an individual observation, with its horizontal position indicating the SHAP value for that feature and observation. RDW, anion gap, blood glucose, pH, WBC, and lactate were identified as the six most influential predictors of TIC prognosis. **Figure 5B** presents an illustrative case analysis demonstrating the model’s prediction process for an individual patient. In this visualization, red indicates positive contributions to the predicted outcome, whereas blue indicates negative contributions. The f(x) value represents the SHAP value associated with each factor. For this specific patient, the logistic classifier model predicted a TIC prognostic risk approximately 20% higher than the baseline value E[f(x)]. The primary contributors to this prediction, based on SHAP values, were RDW, anion gap, and blood glucose.

**Figure 5:**
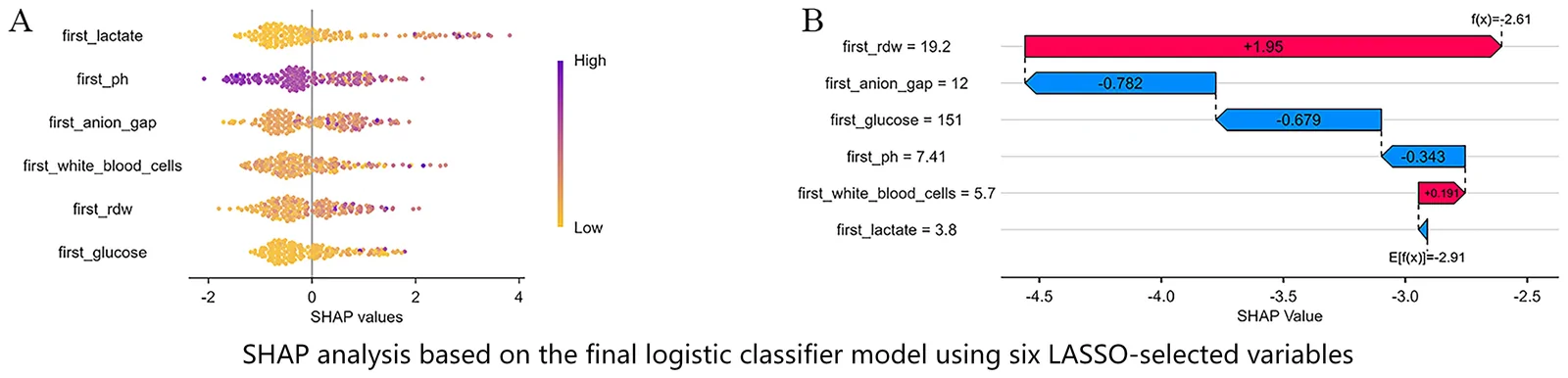
Interpretability analysis of the logistic classifier model. SHAP analysis based on the final logistic classifier model using six LASSO-selected variables. **(A)** SHAP summary beeswarm plot showing the contribution of each feature to the model output. Points are colored by feature value, from low (yellow) to high (purple), with SHAP values displayed on the x‑axis. **(B)** SHAP bar chart illustrating the impact direction and magnitude of each variable on the logistic classifier model output.

### 3.6 GBTM trajectory identification and validation

Latent class models ranging from one to five classes were evaluated (**Supplementary Table 4**). As the number of classes increased, the AIC value decreased, with the lowest AIC observed in the five-class model (AIC = 2828.30). The average posterior probabilities (AvePP) for all models remained consistently high (> 0.70).

During comparison of candidate latent class models, AIC values, AvePP, and subgroup proportions were jointly considered. Although the five-class model yielded the lowest AIC (2828.30), it contained a subgroup with a relatively small sample proportion (5.9%), raising concerns regarding potential overfitting. The three-class model (Class 3) demonstrated a relatively low AIC (2898.23), and the AvePP values for the three classes were 0.9996, 0.9997, and 0.9998, respectively, all exceeding 0.70, indicating excellent classification accuracy.

This model also exhibited a relatively balanced subgroup distribution and a moderate level of model complexity, representing a favorable balance between goodness-of-fit and parsimony. Furthermore, the three trajectory patterns—declining-then-stabilizing, persistently fluctuating, and stabilizing-then-rising—demonstrated clear and clinically interpretable pathophysiological patterns. Based on these considerations, the three-class model was selected as the optimal model for subsequent analyses (**Figure 6A**).

**Figure 6:**
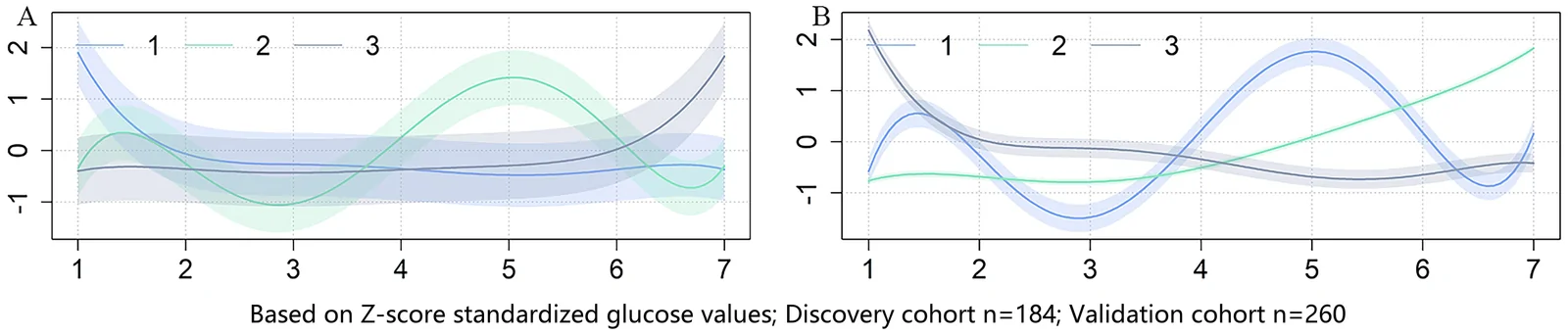
Identification and s validation of blood glucose trajectory subphenotypes using GBTM. Based on Z-score standardized glucose values; Discovery cohort n=184; Validation cohort n=260. **(A)** Trajectory patterns identified in the discovery cohort (n = 184). **(B)** Validation of the trajectory patterns in the external validation cohort (n = 260). Three distinct trajectories are shown with smooth lines and shaded confidence intervals (green, blue, and gray lines).

**Figure 6A** illustrates three distinct glucose trajectory patterns identified using Z-score standardized blood glucose values (the corresponding raw glucose ranges were calculated through inverse transformation of the Z-scores). Class 1 (decreasing-then-stable trajectory) started with relatively high standardized glucose values (approximately 1.5–2.0, corresponding to raw glucose levels of approximately 30–35 mmol/L). Some patients in this class exhibited extreme stress-induced hyperglycemia at baseline, followed by a rapid decline and subsequent stabilization at lower levels (approximately −0.5 to 0, corresponding to raw glucose levels of approximately 7–11 mmol/L). Class 2 (persistently fluctuating trajectory) maintained relatively stable standardized glucose values throughout the 7-day observation period (approximately 0.5–1.0, corresponding to raw glucose levels of approximately 13–14 mmol/L), representing a pattern of sustained moderate-to-severe hyperglycemia. Class 3 (stable-then-increasing trajectory) initially presented with lower glucose levels (approximately −0.5 to 0, corresponding to raw glucose levels of approximately 7–11 mmol/L), followed by a marked increase beginning on days 3–4, reaching higher standardized glucose levels (approximately 1.5–2.0, corresponding to raw glucose levels of approximately 30–35 mmol/L). Overall, Class 1 demonstrated initial improvement followed by stabilization, Class 2 fluctuated within a relatively high range, and Class 3 remained initially stable but subsequently increased during follow-up.

In the local validation cohort, GBTM was applied to classify patients into three trajectory subtypes (**Figure 6B**), and the resulting trajectories were consistent with those observed in the discovery cohort.

To evaluate the impact of data imputation on trajectory stability, we performed a complete-case sensitivity analysis including only patients with fully observed blood glucose measurements (n = 119) (**Supplementary Figure 7**). The optimal model continued to identify three distinct trajectory patterns with nearly identical shapes, and the distribution of patients across trajectory groups remained consistent. The association between the stable-then-increasing trajectory (Class 3) and 28-day mortality remained statistically significant (OR 3.80, 95% CI 2.98–4.85, *p* < 0.001), which was highly consistent with the primary analysis (OR 3.88). These findings demonstrate that our trajectory classification approach was robust to the choice of data imputation strategy and was not substantially affected by the handling of missing longitudinal measurements.

### 3.7 Comparison and validation of baseline characteristics and outcomes of different subphenotypes

A comprehensive comparison of baseline characteristics among the identified subphenotypes (**Supplementary Table 5**) indicated that patients in Class 3 (stabilizing-then-rising) exhibited higher total cholesterol levels and higher mortality rates, whereas patients in Class 2 (persistently fluctuating) demonstrated lower total cholesterol levels and lower mortality rates.

As presented in **Figure 7**, logistic regression analysis (**Figure 7A**) was performed with Class 1 (declining-then-stabilizing) as the reference group after adjustment for age and other baseline variables. Class 2 (persistently fluctuating) was associated with a significantly lower odds ratio for 28-day mortality (OR 0.205, 95% CI 0.102–0.37, *p* < 0.001), corresponding to a 79.5% reduction in mortality risk within 28 days. In contrast, Class 3 (stabilizing-then-rising) demonstrated a significantly higher odds ratio (OR 3.879, 95% CI 3.101–4.886, *p* < 0.001), corresponding to an approximately 289.7% increase in 28-day mortality risk.

**Figure 7:**
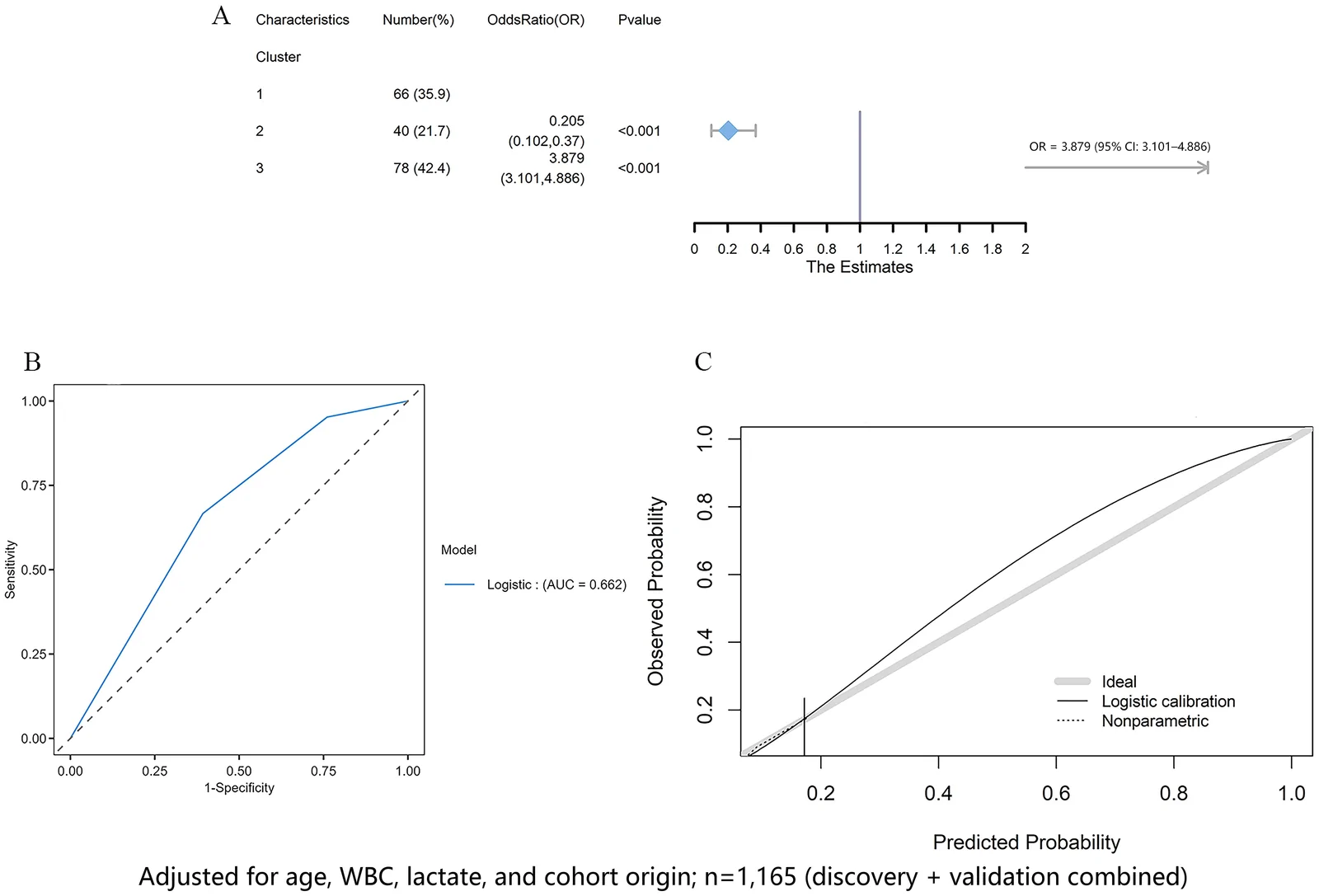
Prognostic performance and external validation of blood glucose trajectory subphenotypes. Adjusted for age, WBC, lactate, and cohort origin; n=1,165 (discovery + validation combined). **(A)** Forest plot and table summarizing odds ratios (OR) and 95% confidence intervals for the three trajectory clusters, adjusted for age, WBC, lactate, and cohort origin (pooled n = 1,165). Class 1 (declining‑then‑stabilizing) served as the reference group. **(B)** Receiver operating characteristic (ROC) curve of the logistic regression model in the external validation cohort, showing an area under the curve (AUC) of 0.662. **(C)** Calibration plot comparing observed versus predicted probabilities for the logistic model, displaying the ideal line, logistic calibration curve, and nonparametric smoothing curve.

**Figure 7B** presents the ROC curves for the logistic regression model applied to the external validation cohort. The horizontal axis represents the false positive rate (1 − specificity), while the vertical axis represents the true positive rate (sensitivity). The model achieved an AUC of 0.662, indicating moderate discrimination between patients experiencing the event and those who did not in the external validation population. The ROC curve was substantially higher than the gray dashed line representing random classification (AUC = 0.5), indicating predictive performance beyond random chance.

Probability calibration curves for the logistic regression model were also generated for the external validation cohort (**Figure 7C**). The calibration curves closely approximated the ideal reference line, with only minor deviations observed in the higher predicted probability range. These findings indicate that the predicted probabilities correspond well with the observed event frequencies in the independent external cohort, demonstrating satisfactory calibration performance.

In the external validation cohort, patients were reclassified into three blood glucose trajectory subphenotypes using GBTM parameters derived from the MIMIC-IV cohort. Clinical outcomes among the subphenotypes (**Table 1**) demonstrated significant differences (*p* < 0.001). Specifically, the persistently fluctuating subtype (subtype 2) exhibited the lowest 28-day mortality (11.11%), whereas the declining-then-stabilizing subtype (subtype 1) and stabilizing-then-rising subtype (subtype 3) demonstrated substantially higher mortality rates (27.27% and 55.00%, respectively). These findings indicate that the persistently fluctuating subtype represents a relatively favorable prognostic phenotype across independent cohorts.

**Table 1:** Clinical characteristics and outcomes of blood glucose trajectory subphenotypes in the local validation cohort.

Variable	1 (n=11)	2 (n=63)	3 (n=20)	p	SMD
wbc	11	63	20		
glu	6.1 (2.9-22.8)	8.9 (4.29-18.45)	12.915 (7.18-28.01)	0.001	0.551
ph	8.02 (3.91-12.25)	8.08 (5.32-12.16)	14.095 (9.8-28.4)	<0.001	1.488
crea	7.35 (7.28-7.57)	7.39 (7.18-7.482)	7.305 (7.12-7.44)	0.002	0.584
TG	2.5 (0.5-7.9)	2.2 (0.4-7.6)	4.45 (0.7-12.9)	<0.001	0.756
TP	143.2 (141.5-145.6)	134.7 (132.8-136.5)	158.7 (157.4-159.9)	<0.001	17.314
BUN	6.5 (6.2-6.8)	5.9 (5.8-5.9)	5.4 (5.3-5.5)	<0.001	6.806
TC	21.8 (19.8-24.1)	16.2 (15.3-17.6)	13.05 (11.8-13.9)	<0.001	6.800
28-day mortality					
Survival	8 (72.73)	56 (88.89)	9 (45.00)	<0.001	0.687
Mortality	3 (27.27)	7 (11.11)	11 (55.00)		

### 3.8 Incremental prognostic value of glucose trajectory classification

To assess the incremental prognostic value of glucose trajectory classification beyond admission glucose and baseline covariates, we compared a baseline model including admission glucose, age, WBC, and lactate with an extended model additionally incorporating trajectory group. Risk reclassification is shown in **Supplementary Figure 8**. The AUC increased from 0.698 to 0.778 (DeLong p = 0.032; **Supplementary Figure 9**). The extended model also showed improved reclassification, with a continuous NRI of 0.650 (95% CI, 0.293–1.000) and an IDI of 0.097 (95% CI, 0.049–0.143) (**Supplementary Figure 10**).

## 4 Discussion

The three dynamic glucose trajectories identified in this study, particularly the stabilizing-then-rising pattern associated with the highest mortality, may reflect differences in the underlying pathophysiological states of patients with TIC. During model selection, the BLRT indicated that increasing the number of trajectory classes from three to four did not significantly improve model fit (*p* = 0.0971). Considering the balanced distribution of the three trajectory groups (proportions ranging from 22.3% to 47.3%) and the excellent classification quality (all AvePP values > 0.999), we confirmed that the three-class model represented the optimal and most robust solution.

Traditionally, identification of this transition relies on frequent invasive coagulation assessments, such as thromboelastography (TEG) or rotational thromboelastometry (ROTEM), which may not be widely available in many clinical settings. In contrast, the stabilizing-then-rising blood glucose trajectory may reflect the core pathological mechanisms underlying this transition, including persistent systemic inflammation and endothelial injury. Delayed and rapidly increasing hyperglycemia following trauma may indicate sustained and inadequately controlled systemic inflammatory response syndrome (SIRS).

This condition is clinically recognized to be associated with coagulation disturbances and adverse outcomes. Previous studies have demonstrated complex interactions between inflammatory responses and coagulation pathways, providing a potential pathophysiological explanation for the observed association between glucose trajectories and TIC-related outcomes (22). In addition, clinical studies have suggested that marked glucose variability may be associated with endothelial dysfunction and altered platelet reactivity; however, these findings are primarily derived from evidence in the fields of cardiovascular disease and diabetes rather than trauma populations (23). In patients with TIC, the direct relationship between glucose variability and dynamic changes in coagulation phenotypes remains to be further elucidated. Consequently, the stabilizing-then-rising trajectory may represent a metabolic manifestation of the pathological cycle involving persistent inflammation, endothelial damage, and coagulation activation, thereby demonstrating an independent association with adverse clinical outcomes.

Notably, higher total cholesterol levels were observed in the stable-then-increasing trajectory subgroup. This finding may be associated with post-traumatic metabolic stress. Severe trauma can induce excessive activation of the HPA axis and the sympathetic nervous system, leading to substantial alterations in systemic metabolism, including disturbances in glucose and lipid metabolism. Clinical studies have demonstrated that trauma-induced stress hyperglycemia is often accompanied by abnormalities in lipid profiles, and these metabolic alterations may jointly reflect a state of systemic metabolic dysregulation under severe stress. The coexistence of multiple metabolic disturbances may further contribute to an increased risk of organ dysfunction.

Post-traumatic stress hyperglycemia is an adaptive metabolic response of the body to severe injury (24, 25). The liver is metabolically reprogrammed to regenerate glucose from lactate and gluconeogenic amino acids to meet the energy demands of immune cells and repairing tissues. However, persistent hyperglycemia can lead to detrimental effects. Previous studies have confirmed that admission hyperglycemia is an independent predictor of coagulopathy in trauma patients (OR = 1.99) (26), and prehospital stress hyperglycemia is significantly associated with in-hospital mortality (aOR = 2.9) (27). Meanwhile, acute hyperglycemia can aggravate trauma-induced endothelial cell injury and glycocalyx shedding and is associated with poor prognosis in trauma patients (28).

It should be emphasized that this study was a retrospective prognostic analysis based on clinical data, aiming to identify prognostic subtypes of TIC through glucose trajectories rather than to investigate specific molecular pathophysiological mechanisms. We did not measure inflammatory biomarkers (e.g. TNF-α and IL-6), endothelial injury markers (e.g. glycocalyx components and von Willebrand factor), coagulation-related molecular markers (e.g. thrombin–antithrombin complexes), or specific lipid metabolites (e.g. sphingolipids and ceramides). Therefore, any potential molecular mechanisms discussed above should be regarded as speculative hypotheses derived from existing literature rather than conclusions directly supported by the present data. These hypotheses require further validation in specifically designed prospective mechanistic studies.

The coexistence of elevated glucose trajectories and increased cholesterol levels observed in this study may therefore indicate a broader metabolic–coagulation dysregulation syndrome, providing an additional perspective beyond conventional coagulation parameters for understanding TIC heterogeneity. These findings challenge the traditional view of hyperglycemia solely as an indicator of stress severity and instead indicate that glucose trajectories may serve as an alternative marker reflecting the intrinsic pathophysiological dynamics of TIC, offering a novel approach for identifying glucose trajectory phenotypes in the absence of real-time coagulation monitoring.

The data-driven analytical framework applied in this study may provide a complementary strategy for early risk stratification in TIC and highlights the potential role of CGM in trauma management. Conventional single-point glucose measurements may fail to detect up to 40% of cases with abnormal glucose metabolism, whereas longitudinal monitoring enables the identification of trajectory-based subgroups that may require differentiated management strategies (29). For example, patients with a stabilizing-then-rising trajectory may benefit from early combined interventions targeting both glucose control and lipid metabolism, such as insulin therapy and lipid-lowering agents (e.g., statins) (17). Additionally, these patients may require increased surveillance for delayed thrombotic complications, including deep vein thrombosis, pulmonary embolism, and multiple organ dysfunction syndrome (MODS).

In contrast, patients exhibiting persistently fluctuating glucose trajectories may experience disruption of metabolic homeostasis if glucose-lowering interventions are overly aggressive (30). Future management strategies may therefore need to extend beyond glycemic control alone, integrating anti-inflammatory therapies, endothelial protective strategies, and targeted anticoagulation approaches.

The limitations of the present study provide several directions for future research. First, prospective cohort studies incorporating continuous coagulation monitoring methods, such as TEG or ROTEM, are needed to directly evaluate the relationship between blood glucose trajectories and dynamic changes in coagulation phenotypes. Second, targeted metabolomic or proteomic analyses may help identify the biomarker networks underlying different trajectory subtypes, thereby providing potential molecular targets for precision therapy.

Model calibration in this study was evaluated using both calibration curves and ECE. Compared with the traditional Hosmer–Lemeshow test, this approach offers several methodological advantages. Calibration curves provide a visual representation of model performance across the entire spectrum of predicted risk, enabling clinicians to more clearly evaluate prediction accuracy across different risk levels. Furthermore, ECE quantifies calibration error as a continuous metric rather than relying on a binary statistical significance threshold. This approach also reduces the sensitivity of the Hosmer–Lemeshow test to arbitrary grouping decisions. Although the model demonstrated a slight underestimation of risk in the highest-risk range, overall calibration performance remained acceptable (ECE = 0.119) and within clinically acceptable limits (31).

Several limitations should be acknowledged.

First, the retrospective design introduces the possibility of information bias, including potential inaccuracies in recorded data and missing variables.

Second, limitations inherent to the database structure may have resulted in the exclusion of certain clinically relevant variables, which may affect the comprehensiveness of the predictive model.

Third, although external validation was performed using data from Hebei Medical University Third Hospital, the validation dataset originated from a single center, and additional large-scale prospective studies are required to confirm the model’s accuracy and generalizability across diverse populations.

Fourth, despite external validation, the validation cohort remained relatively small and differed from the discovery cohort in terms of patient characteristics and treatment practices. These differences may contribute to variations in absolute mortality risk estimates across subphenotypes between cohorts. Our sensitivity analyses demonstrated that the model based on the six LASSO-selected variables achieved nearly identical discriminative performance compared with the full-feature model incorporating all available variables. This finding has important practical implications, suggesting that TIC prognosis is primarily driven by a core set of metabolic and acid–base disturbance indicators (reflected by glucose, lactate, pH, and anion gap), together with markers of inflammatory and hematological stress responses (reflected by white blood cell count and RDW). Although complex machine learning algorithms are theoretically capable of capturing higher-order interactions among numerous variables, the addition of extra clinical features provided minimal incremental benefit in our dataset. These findings support the clinical utility of a focused and readily obtainable biomarker combination for early risk stratification, particularly in resource-limited or time-sensitive clinical settings where comprehensive laboratory assessments may not be immediately available. Although statistical adjustments were applied by incorporating cohort origin as a covariate, and the reliability of the findings was supported, this approach primarily addresses measured confounders. Future research should therefore evaluate the generalizability of the identified trajectory subtypes in larger and more diverse prospective populations.

Fifth, because the retrospective datasets were derived from different healthcare systems, the scope of baseline data collection differed between cohorts. Although this discrepancy did not affect the core variables included in the final predictive model, it may have limited the analysis of certain secondary biomarkers in the validation cohort.

Sixth, it should be emphasized that this study did not measure inflammatory factors, endothelial function markers, or specific coagulation molecular markers. The above discussion regarding the association between TIC phenotype evolution and blood glucose trajectory is merely a speculative hypothesis based on trauma literature and clinical observations, rather than a conclusion directly proven by the data from this study. Future research should combine continuous glucose monitoring with continuous viscoelastic coagulation testing to directly evaluate the relationship between blood glucose trajectory and dynamic changes in coagulation phenotypes.

Seventh, this study adopts INR ≥ 1.5 or APTT ≥ 35 seconds as the definition criteria for TIC, a method widely used in the field of TIC research. However, we acknowledge the inherent limitations of this definition. Traditional coagulation tests, such as PT, INR, APTT, have significant operational and mechanistic limitations in acute trauma settings (32). These tests typically require 45–60 minutes from blood collection to results, failing to meet the timeliness requirements of trauma resuscitation. Additionally, their reliability is further reduced in trauma patients with hypothermia and acidosis. Moreover, they only provide a “static snapshot” of the coagulation process and cannot assess dynamic clot formation, platelet function, or fibrinolysis. Research has clearly indicated that isolated INR abnormalities do not truly reflect coagulation dysfunction. In contrast, viscoelastic hemostatic assays (VHAs), primarily including TEG and ROTEM, can provide a rapid and comprehensive assessment of clot initiation, clot strength, and stability within 10–15 minutes, enabling functional characterization of TIC. VHAs can detect abnormalities that conventional coagulation tests cannot identify, including prolonged clot formation time, platelet and fibrinogen dysfunction, hyperfibrinolysis, etc. More importantly, TIC encompasses a bidirectional phenotypic spectrum of hypercoagulation and hypocoagulation, while VHAs can identify and individually treat different coagulation phenotypes within this dynamic spectrum. Multicenter studies have confirmed that TIC scores based on TEG/ROTEM are independently associated with in-hospital mortality and red blood cell transfusion volume. This study is limited by its retrospective design, as TEG/ROTEM results were not available, so the definition using INR/APTT was adopted. However, the limitations of the INR/APTT definition precisely support the rationale of using the GBTM method to identify TIC subtypes because static indices cannot capture the dynamic heterogeneity of TIC, we need to use the dynamic marker of glucose trajectory to indirectly reflect the phenotypic evolution of TIC. Future research should combine continuous glucose monitoring with viscoelastic tests such as TEG/ROTEM to more comprehensively characterize the dynamic phenotypic evolution of TIC, and to verify the direct association between glucose trajectory subtypes and coagulation phenotypes.

Eighth, while we applied sensitivity analyses to confirm the robustness of our imputation strategy, we acknowledge that our primary imputation approach did not explicitly model the temporal autocorrelation inherent in serial glucose measurements. Given the relatively low missing rate and the consistency of the complete-case analysis, we believe this limitation is unlikely to have substantially affected our main conclusions. Nevertheless, future prospective studies employing continuous glucose monitoring with standardized high-frequency sampling would enable more sophisticated time-series imputation, such as Kalman filtering, to capture intra-day variability and autocorrelation more precisely.

Ninth, the trajectory analysis cohort represented only 20.3% of the overall TIC population (184/905), primarily due to the irregular timing of glucose measurements in the MIMIC-IV database, which prevented many patients from generating a complete 7-day standardized morning glucose profile. Although we compared the baseline characteristics between included and excluded patients and found no significant differences in observable variables, we cannot completely exclude potential selection bias caused by unmeasured confounders, such as ICU treatment intensity and nursing practices. Therefore, the identified glucose trajectory phenotypes and their associations with outcomes should be interpreted primarily as findings within the TIC subgroup with complete morning glucose monitoring data. The generalizability of these findings to the broader TIC population requires further validation in future prospective studies.

Tenth, although the trajectory models adjusted for multiple baseline clinical variables, this retrospective study could not fully account for differences in ICU management after admission. Interventions such as insulin therapy, nutritional support, transfusion strategies, vasopressor use, corticosteroid administration, and other critical care treatments may influence glucose trajectories as well as clinical outcomes. Because detailed information on the timing, intensity, and longitudinal changes of these interventions was not uniformly available, residual confounding cannot be excluded. Future prospective studies incorporating standardized treatment protocols or time-varying treatment data are needed to further clarify the independent prognostic significance of glucose trajectory phenotypes.

Future prospective studies using standardized data collection protocols may further validate and expand these findings. Finally, although several key confounding variables, including age, ISS, WBC, and lactate, were adjusted for in the analysis, retrospective studies remain susceptible to unmeasured confounding factors such as undocumented medication exposure, microcirculatory status, or genetic predisposition. Consequently, the observed association between blood glucose trajectories and adverse outcomes may be partially influenced by unmeasured variables, and residual confounding cannot be completely excluded. Nonetheless, the proposed model may provide practical clinical value by assisting critical care clinicians in the early identification of high-risk patients with TIC and facilitating timely clinical intervention.

## Data Availability

The raw data supporting the conclusions of this article will be made available by the authors, without undue reservation.
