# Supplementary material for: Machine learning–based prognostic prediction and subphenotype identification in trauma-induced coagulopathy using early blood glucose trajectories
Source: Front Endocrinol (Lausanne). 2026 Sep 15;17:1839373. doi: 10.3389/fendo.2026.1839373 (PMC13619434; doi:10.3389/fendo.2026.1839373)
Supplement: Supplementary file 1 [file DataSheet1.docx]

**Supplementary Table 1. Baseline characteristics of the MIMIC discovery cohort**

| **Variable** | **Total (*n* = 905)** | **Survivors (*n* = 693)** | **Mortalities (*n* = 212)** | ***p*-value** |
| --- | --- | --- | --- | --- |
| Age | 59.75 (15.42) | 60.22 (15.02) | 58.20 (16.59) | 0.115 |
| Weight (kg) | 85.69 (25.62) | 86.13 (25.59) | 84.26 (25.71) | 0.355 |
| Height (cm) | 170.66 (10.33) | 171.06 (10.28) | 169.33 (10.43) | 0.035 |
| Heart rate (beats/min) | 93.65 (21.26) | 92.07 (20.44) | 98.83 (23.04) | <0.001 |
| Respiratory rate (breaths/min) | 19.55 (6.63) | 19.13 (6.33) | 20.92 (7.41) | 0.002 |
| Oxygen saturation (%) | 96.42 (5.12) | 96.75 (4.50) | 95.35 (6.67) | 0.005 |
| Temperature (℉) | 97.23 (8.06) | 98.01 (4.29) | 94.70 (14.48) | 0.001 |
| Noninvasive systolic blood pressure (mmHg) | 116.47 (26.51) | 118.26 (25.38) | 110.61 (29.21) | <0.001 |
| Noninvasive diastolic blood pressure (mmHg) | 68.42 (20.45) | 69.11 (20.33) | 66.16 (20.70) | 0.069 |
| Mean noninvasive blood pressure (mmHg) | 80.59 (20.69) | 81.49 (20.60) | 77.64 (20.76) | 0.018 |
| Arterial systolic blood pressure (mmHg) | 118.29 (29.34) | 120.38 (28.78) | 111.46 (30.18) | <0.001 |
| Arterial diastolic blood pressure (mmHg) | 60.88 (19.63) | 61.06 (17.07) | 60.31 (26.34) | 0.698 |
| Mean arterial blood pressure (mmHg) | 72.35 (40.12) | 72.00 (38.54) | 73.49 (44.95) | 0.662 |
| Absolute lymphocyte count (K/μL) | 1.23 (1.23) | 1.17 (1.04) | 1.40 (1.71) | 0.069 |
| Hematocrit (%) | 29.80 (7.31) | 29.59 (6.96) | 30.49 (8.35) | 0.153 |
| Hemoglobin (g/dL) | 9.73 (2.43) | 9.68 (2.33) | 9.89 (2.74) | 0.296 |
| Platelet count (K/μL) | 165.03 (112.22) | 170.75 (117.47) | 146.32 (90.77) | 0.002 |
| RDW (%) | 16.11 (2.81) | 15.92 (2.63) | 16.73 (3.26) | 0.001 |
| Red blood cell count (m/μL) | 3.21 (0.86) | 3.20 (0.83) | 3.22 (0.95) | 0.793 |
| White blood cell count (m/μL) | 12.31 (9.03) | 11.56 (8.65) | 14.77 (9.81) | <0.001 |
| Absolute neutrophil count (K/μL) | 10.11 (7.00) | 9.63 (6.69) | 11.68 (7.74) | <0.001 |
| Glycosylated hemoglobin (%) | 6.22 (1.81) | 6.17 (1.79) | 6.39 (1.85) | 0.131 |
| Albumin (g/dL) | 2.93 (0.67) | 2.98 (0.64) | 2.78 (0.74) | <0.001 |
| Anion gap (mEq/L) | 15.83 (5.86) | 14.74 (5.02) | 19.39 (6.93) | <0.001 |
| Total calcium (mg/dL) | 8.32 (1.24) | 8.24 (0.97) | 8.58 (1.85) | 0.011 |
| Chloride (mEq/L) | 103.00 (7.89) | 103.10 (7.62) | 102.67 (8.70) | 0.511 |
| Globulin (g/dL) | 2.62 (0.86) | 2.58 (0.81) | 2.78 (0.98) | 0.007 |
| Blood glucose (mg/dL) | 158.29 (106.74) | 146.40 (90.41) | 197.17 (141.52) | <0.001 |
| Potassium (mEq/L) | 4.28 (0.89) | 4.23 (0.84) | 4.46 (1.05) | 0.003 |
| Total protein (g/dL) | 5.66 (1.15) | 5.68 (1.12) | 5.58 (1.24) | 0.281 |
| Sodium (mEq/L) | 137.77 (6.50) | 137.52 (5.97) | 138.61 (7.96) | 0.065 |
| D-dimer (ng/mL) | 4,620.79 (4,927.25) | 4,452.56 (4,823.06) | 5,170.72 (5,227.61) | 0.076 |
| Functional fibrinogen (mg/dL) | 294.02 (193.42) | 304.42 (192.92) | 260.02 (191.56) | 0.003 |
| International normalized ratio (INR) | 2.34 (1.23) | 2.29 (1.18) | 2.50 (1.37) | 0.042 |
| Prothrombin time (PT, sec) | 24.75 (13.41) | 24.31 (12.91) | 26.20 (14.87) | 0.096 |
| Thrombin time (sec) | 43.31 (47.44) | 41.63 (46.27) | 48.79 (50.83) | 0.068 |
| Calculated total CO₂ (mEq/L) | 23.58 (6.37) | 24.37 (6.04) | 20.98 (6.73) | <0.001 |
| Free calcium (mmol/L) | 1.09 (0.16) | 1.09 (0.15) | 1.08 (0.17) | 0.390 |
| Lactate (mmol/L) | 3.46 (3.38) | 2.73 (2.32) | 5.85 (4.86) | <0.001 |
| Partial pressure of carbon dioxide (mmHg) | 44.17 (14.07) | 43.33 (12.54) | 46.92 (17.98) | 0.007 |
| pH | 7.32 (0.13) | 7.35 (0.10) | 7.25 (0.16) | <0.001 |
| Partial pressure of oxygen (mmHg) | 132.37 (113.50) | 129.49 (108.62) | 141.75 (127.99) | 0.208 |
| Alanine aminotransferase (ALT, IU/L) | 246.75 (934.01) | 194.29 (809.35) | 418.24 (1,245.27) | 0.014 |
| Aspartate aminotransferase (AST, IU/L) | 357.96 (1,153.37) | 275.50 (1,006.91) | 627.51 (1,509.60) | 0.002 |
| Direct bilirubin (mg/dL) | 2.75 (4.07) | 2.52 (3.75) | 3.51 (4.94) | 0.007 |
| Indirect bilirubin (mg/dL) | 1.71 (2.46) | 1.67 (2.55) | 1.82 (2.13) | 0.415 |
| Total bilirubin (mg/dL) | 2.99 (5.41) | 2.70 (4.81) | 3.92 (6.96) | 0.018 |
| Creatinine (mg/dL) | 1.61 (1.43) | 1.55 (1.47) | 1.80 (1.29) | 0.016 |
| Blood urea nitrogen (mg/dL) | 29.26 (24.00) | 28.02 (22.81) | 33.29 (27.20) | 0.011 |
| Uric acid (mg/dL) | 5.91 (3.52) | 5.72 (3.44) | 6.55 (3.69) | 0.004 |
| Creatine kinase (CK, IU/L) | 2,264.12 (10,820.82) | 1,860.46 (10,393.95) | 3,583.64 (12,044.13) | 0.061 |
| Lactate dehydrogenase (LDH, IU/L) | 642.11 (1,444.46) | 546.13 (1,386.05) | 955.88 (1,584.11) | <0.001 |
| NT-proBNP (pg/mL) | 6,403.52 (8,364.61) | 6,258.13 (8,331.20) | 6,878.78 (8,475.33) | 0.350 |
| Troponin T (ng/mL) | 0.50 (1.80) | 0.44 (1.71) | 0.70 (2.05) | 0.089 |
| Urine albumin (mg/dL) | 39.27 (77.62) | 41.19 (81.37) | 32.97 (63.57) | 0.125 |
| Urine albumin/creatinine ratio | 487.26 (1,005.53) | 485.10 (1,017.05) | 494.31 (969.23) | 0.905 |
| High-density lipoprotein cholesterol (mg/dL) | 31.07 (16.79) | 31.78 (17.20) | 28.73 (15.21) | 0.014 |
| Calculated low-density lipoprotein cholesterol (mg/dL) | 61.23 (32.83) | 61.12 (32.40) | 61.58 (34.28) | 0.860 |
| Total cholesterol (mg/dL) | 116.01 (48.17) | 114.59 (43.33) | 120.67 (61.29) | 0.180 |
| Triglycerides (mg/dL) | 185.41 (226.82) | 173.62 (168.08) | 223.95 (354.70) | 0.047 |
| Sex |  | | | 0.047 |
| Female | 328.00 (36.24%) | 239.00 (34.49%) | 89.00 (41.98%) |  |
| Male | 577.00 (63.76%) | 454.00 (65.51%) | 123.00 (58.02%) |  |

**Supplementary Table 2. Baseline characteristics of the local validation cohort**

| Variable | Total (*n* = 260) | Survivors (*n* = 214) | Mortalities (*n* = 46) | *p* |
| --- | --- | --- | --- | --- |
| Age | 56.5 (8-85) | 55 (8-76) | 63 (9-85) | 0.003 |
| Temperature (°C) | 36.5 (35.4-40.8) | 36.5 (35.4-39) | 36.5 (35.5-40.8) | 0.709 |
| Respiratory rate (breaths/min) | 20 (10-40) | 20 (10-40) | 20 (13-36) | 0.740 |
| Heart rate (beats/min) | 90 (25-150) | 90 (25-150) | 97 (53-143) | 0.315 |
| Mean arterial pressure (mmHg) | 94 (47.333-129.667) | 94.667 (54.333-129.667) | 91.5 (47.333-126) | 0.142 |
| White blood cell count (×10⁹/L) | 10.87 (2.9-28.08) | 10.58 (2.9-24.75) | 12.955 (4.53-28.08) | <0.001 |
| Hemoglobin (g/L) | 101 (4.47-174) | 101.5 (4.47-174) | 94.5 (63-146) | 0.590 |
| RDW (%) | 16.38 (14.96-18.97) | 16.355 (14.96-18.97) | 16.43 (15.63-17.68) | 0.886 |
| Blood glucose (mmol/L) | 8.525 (3.55-28.4) | 8.36 (3.79-28.4) | 10.59 (3.55-26.09) | <0.001 |
| pH | 7.4 (7.1-7.57) | 7.4 (7.18-7.53) | 7.35 (7.1-7.57) | <0.001 |
| Lactate (mmol/L) | 1.7 (0.2-12.9) | 1.6 (0.2-8.7) | 2.7 (0.4-12.9) | <0.001 |
| Anion gap | 15.03 (13.32-20.53) | 15.035 (13.32-20.53) | 14.855 (13.32-20.32) | 0.560 |
| Sex |  |  |  |  |
| 1 | 189 (72.69) | 152 (71.03) | 37 (80.43) | 0.264 |
| 2 | 71 (27.31) | 62 (28.97) | 9 (19.57) |  |

A total of 260 patients from Hebei Medical University Third Hospital were included in the study, of whom 214 survived and 46 died. Compared with the mortality group, the survival group was relatively younger and had higher pH values but lower heart rate, white blood cell count, albumin levels, blood glucose, and lactate levels.

**Supplementary Table 3. Performance comparison of twelve machine learning models**

| **Model** | Accuracy | AUROC | \|Specificity | Sensitivity | FNR | FPR |
| --- | --- | --- | --- | --- | --- | --- |
| **RFTEST** | 0.779 | 0.786 | 0.681 | 0.947 | 0.687 | 0.052 |
| **DesicionTreeTEST** | 0.779 | 0.726 | 0.722 | 0.962 | 0.729 | 0.037 |
| **GBDTTEST** | 0.707 | 0.623 | 0.441 | 0.819 | 0.604 | 0.187 |
| **AdaBoostTEST** | 0.701 | 0.617 | 0.437 | 0.796 | 0.562 | 0.203 |
| **LGBMTEST** | 0.751 | 0.748 | 0.542 | 0.879 | 0.604 | 0.120 |
| **LogisticTEST** | 0.795 | 0.785 | 0.739 | 0.954 | 0.645 | 0.045 |
| **NBTEST** | 0.712 | 0.685 | 0.464 | 0.774 | 0.458 | 0.225 |
| **CatBoostTEST** | 0.784 | 0.786 | 0.695 | 0.947 | 0.666 | 0.052 |
| **XGBTEST** | 0.745 | 0.745 | 0.531 | 0.887 | 0.645 | 0.112 |
| **SVMTEST** | 0.740 | 0.795 | 0.666 | 0.992 | 0.958 | 0.007 |
| **MLPTEST** | 0.801 | 0.747 | 0.750 | 0.954 | 0.625 | 0.045 |
| **KNNCTEST** | 0.756 | 0.719 | 0.583 | 0.924 | 0.708 | 0.075 |
| **mean_scores** | 0.754 | 0.730 | 0.604 | 0.903 | 0.657 | 0.096 |

**Supplementary Table 4. Model fit and performance comparison of GBTM models with one to five classes**

| **MODEL** | **AvePP** | | | | | **AIC** |
| --- | --- | --- | --- | --- | --- | --- |
|  | **C1** | **C2** | **C3** | **C4** | **C5** |  |
| 1 | 1（100%） | NA | NA | NA | NA | 3893.9809 |
| 2 | 0.9937（40.5%） | 0.9999（59.5%） | NA | NA | NA | 3294.5258 |
| 3 | 0.9996（35.9%） | 0.9997（21.7%） | 0.9998（42.4%） | NA | NA | 2898.2275 |
| 4 | 0.9997（40.5%） | 0.9999（20%） | 0.9116（10.5%） | 0.9536（29%） | NA | 2894.1848 |
| 5 | 0.909（5.9%） | 1（20%） | 0.954（34.6%） | 0.913（10.4%） | 0.952（29.1%） | 2828.3019 |

**Supplementary Table 5. Baseline characteristics of blood glucose trajectory subphenotypes**

| **Feature** | 1（n=66） | 2(n=40) | 3(n=78) | p | SMD |
| --- | --- | --- | --- | --- | --- |
| **White blood cell count** | 11.7 (2-29) | 10.9 (3.1-54.3) | 9 (2.5-55.5) | 0.962 | 0.035 |
| **Blood lipids** | 132.5 (34-671) | 133.5 (62-671) | 121 (35-641) | 0.696 | 0.098 |
| **Total protein** | 6.8 (4.6-11.1) | 5.8 (5-6.2) | 5.4 (4.7-6.1) | 0.084 | 0.859 |
| **Blood urea nitrogen** | 21 (10-39) | 15 (6-23) | 12 (9-26) | 0.043 | 0.823 |
| **Total cholesterol** | 142 (35-176) | 85.5 (69-156) | 159 (131-179) | 0.016 | 1.333 |
| **28-day mortality** |  | | | 0.034 | 0.358 |
| **Survival** | 60 (90.91) | 39 (97.50) | 64 (82.05) |  |  |
| **Mortality** | 6 (9.09) | 1 (2.50) | 14 (17.95) |  |  |
